# Inflammation induced by tumor-associated nerves promotes resistance to anti-PD-1 therapy in cancer patients and is targetable by interleukin-6 blockade

**DOI:** 10.21203/rs.3.rs-3161761/v1

**Published:** 2023-07-18

**Authors:** Erez N. Baruch, Priyadahrshini Nagarajan, Frederico O. Gleber-Netto, Xiayu Rao, Tongxin Xie, Shamima Akhter, Adebayo Adewale, Islam Shajedul, Brandi J Mattson, Renata Ferrarotto, Michael K. Wong, Michael A Davies, Sonali Jindal, Sreyashi Basu, Catherine Harwood, Irene Leigh, Nadim Ajami, Andrew Futreal, Micah Castillo, Preethi Gunaratne, Ryan P. Goepfert, Nikhil Khushalani, Jing Wang, Stephanie Watowich, George A Calin, Michael R. Migden, Paola Vermeer, Nisha D’Silva, Dan Yaniv, Jared K Burks, Javier Gomez, Patrick M Dougherty, Kenneth Y. Tsai, James P Allison, Padmanee Sharma, Jennifer Wargo, Jeffrey N. Myers, Neil D. Gross, Moran Amit

**Affiliations:** 1 Division of Cancer Medicine, Hematology and Oncology Fellowship program, the University of Texas MD Anderson Cancer Center, Houston, TX, USA.; 2 Department of Pathology, the University of Texas MD Anderson Cancer Center, Houston, TX, USA.; 3 Department of Head and Neck Surgery, the University of Texas MD Anderson Cancer Center, Houston, TX, USA.; 4 Department of Bioinformatics and Computational Biology, Division of Basic Sciences, The University of Texas MD Anderson Cancer Center, Houston, TX, USA.; 5 The Neurodegeneration Consortium, Therapeutics Discovery Division, University of Texas MD Anderson Cancer Center, Houston, TX, USA.; 6 Department of Head and Neck Thoracic Medical Oncology, The University of Texas MD Anderson Cancer Center, Houston, TX, USA.; 7 Department of Melanoma Medical Oncology, Division of Cancer Medicine, the University of Texas MD Anderson Cancer Center, Houston, TX, USA.; 8 Department of Immunology, Division of Cancer Medicine, The University of Texas MD Anderson Cancer Center, Houston, TX, USA.; 9 Department of Dermatology, Royal London Hospital, Barts Health NHS Trust, Centre for Cell Biology and Cutaneous Research, Blizard Institute Barts and the London School of Medicine and Dentistry Queen Mary University of London, UK.; 10 Department of Genomic Medicine, Division of Cancer Medicine, the University of Texas MD Anderson Cancer Center, Houston, TX, USA.; 11 Department of Biology and Biochemistry, University of Houston Sequencing and Gene Editing Core, University of Houston, Houston, TX, USA.; 12 Department of Tumor Biology, Moffitt Cancer Center, Tampa, FL, USA.; 13 Department of Translational Molecular Pathology, the University of Texas MD Anderson Cancer Center, Houston, TX, USA.; 14 Department of Dermatology, the University of Texas MD Anderson Cancer Center, Houston, TX, USA.; 15 Cancer Biology and Immunotherapies Group, Sanford Research, Sioux Falls, SD, USA.; 16 Department of Dentistry & Pathology, the University of Michigan, Ann Arbor, MI, USA.; 17 Department of Leukemia, Division of Cancer Medicine, the University of Texas MD Anderson Cancer Center, Houston, TX, USA.; 18 Department of Pain Medicine, Division of Anesthesiology, Critical Care, and Pain Medicine, the University of Texas MD Anderson Cancer Center, Houston, TX, USA.; 19 Department of Surgical Oncology, the University of Texas MD Anderson Cancer Center, Houston, TX, USA.; 20 The University of Texas MD Anderson Cancer Center UTHealth Graduate School of Biomedical Sciences, Houston, TX

## Abstract

While the nervous system has reciprocal interactions with both cancer and the immune system, little is known about the potential role of tumor associated nerves (TANs) in modulating anti-tumoral immunity. Moreover, while peri-neural invasion is a well establish poor prognostic factor across cancer types, the mechanisms driving this clinical effect remain unknown. Here, we provide clinical and mechniastic association between TANs damage and resistance to anti-PD-1 therapy. Using electron microscopy, electrical conduction studies, and tumor samples of cutaneous squamous cell carcinoma (cSCC) patients, we showed that cancer cells can destroy myelin sheath and induce TANs degeneration. Multi-omics and spatial analyses of tumor samples from cSCC patients who underwent neoadjuvant anti-PD-1 therapy demonstrated that anti-PD-1 non-responders had higher rates of peri-neural invasion, TANs damage and degeneration compared to responders, both at baseline and following neoadjuvant treatment. Tumors from non-responders were also characterized by a sustained signaling of interferon type I (IFN-I) – known to both propagate nerve degeneration and to dampen anti-tumoral immunity. Peri-neural niches of non-responders were characterized by higher immune activity compared to responders, including immune-suppressive activity of M2 macrophages, and T regulatory cells. This tumor promoting inflammation expanded to the rest of the tumor microenvironment in non-responders. Anti-PD-1 efficacy was dampened by inducing nerve damage prior to treatment administration in a murine model. In contrast, anti-PD-1 efficacy was enhanced by denervation and by interleukin-6 blockade. These findings suggested a potential novel anti-PD-1 resistance drived by TANs damage and inflammation. This resistance mechanism is targetable and may have therapeutic implications in other neurotropic cancers with poor response to anti-PD-1 therapy such as pancreatic, prostate, and breast cancers.

## Introduction

The development of cancer immunotherapy, specifically that of programmed cell death 1 (PD-1) blocking antibodies, has ushered in a new era in oncological care. Anti-PD-1 therapy has induced profound and durable tumor regression in specific patient subsets across multiple cancer types^1^. Yet, the majority of patients still do not respond to anti-PD-1 treatment^2–5^. Colossal efforts have been invested in identifying potential resistance mechanisms to anti-PD-1 therapy. CD8^+^ T cell activity within the tumor microenvironment (TME), a key effector of anti-tumor immune activity^6,7^, has been extensively studied, leading to discoveries of new immune checkpoints and immunotherapies^8,9^. However, elimination of cancer cells by CD8^+^ T cells is only the final chord in an intricate symphony. To migrate into a tumor, become activated, proliferate, and resist exhaustion, CD8^+^ T cells must interact with not only cancer cells but also multiple other immune cells in the TME that regulate T cell activity^10^. Moreover, the associations of fibroblasts^11^ and intra-tumoral bacteria^12^ with clinical response to anti-PD-1 therapy suggest that other, non-immune residents of the TME may also regulate the antitumoral immune response.

Tumor-associated nerves (TANs) are peripheral nerves fibers found within or in close proximity to tumors^13–15^. TANs may promote tumor progression via adrenergic signaling^16^. Tumor infiltration into TANs, known as perineural invasion (PNI), is a well-established adverse prognostic factor in cancer^17,18^, especially in cutaneous squamous cell carcinoma (cSCC)^19^. However, little is known about the role of TANs in regulating anti-tumoral immune activity^20^. This limited knowledge contrasts with the established evidence of bidirectional communication between the peripheral nervous system (PNS) and immune system. The PNS supports hematopoiesis, regulates immune responses against infections, and participates in the creation of immune memory^21–23^. Injured peripheral nerves attract immune cells such as M2 macrophages^24–26^ – key players in tumor progression^27,28^ and resistance to anti-PD-1 therapy^29^ – to promote nerve healing and regeneration^24,30^. Yet, the immune-nerve-cancer reciprocal relationship remains largely uncharted. Here, we delineated the intra-tumoral immune and neural phenotypes among cSCC patients who underwent anti-PD-1 therapy and demonstrate the role of TANs in resistance to anti-PD-1 therapy.

## Results

### Cancer-induced nerve damage is associated with poor clinical response to anti-PD-1 therapy.

**Figure 1.**
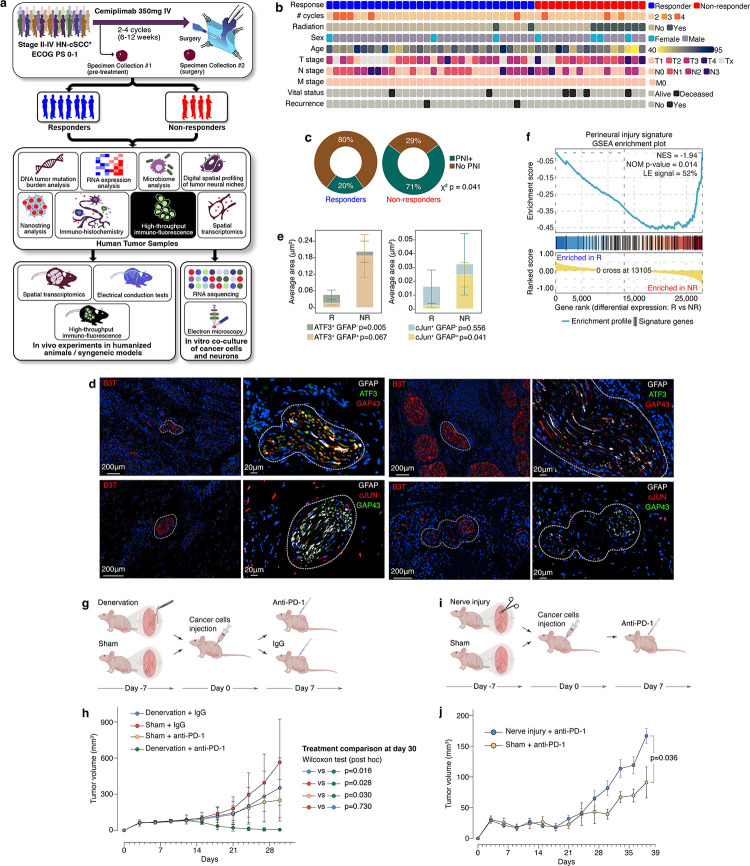
Nerve damage is associated with resistance to anti-PD-1 therapy. **(a)** Overview of the study workflow. Tumor samples from two anti-PD-1 neoadjuvant clinical trials for cutaneous squamous cell carcinoma (cSCC, NCT03565783 and NCT04154943) were used for molecular analysis. Findings were validated using in vivo and intro models. **(b)** Clinical characteristics of the patients from our clinical trial cohorts. R, Responders to anti-PD-1 treatment, NR, non-responders; Tumor (T), Nodal (N), and Metastases (M) status were determined based on the AJCC 8th edition^50^. **(c)** Peri-neural invasion rates in the clinical trial tumor samples according to response status **(d)** Representative images demonstrating expression of neural injury markers ATF3 and cJUN in tumor-associated nerves (TANs), with their distribution among neural (GFAP^−^) and Schwan (GFAP^+^) components **(e)** Histograms of mean ± SEM ATF3 and cJUN expression levels according to response status. **(f)** Gene Set Enrichment Analysis (GSEA) of genes assocated with TANs proximity to cancer cells^40^; negative Normalized Enrichment Score (NES) was −1.94. **(g)** Denervation (nerve elimination) mouse experiment design; biweekly anti-mouse PD-1 treatment was administered 7 days after tumor implantation. **(h)** Tumor growth plot (day 30) of the devenervation mouse experiment; please refer to Supplementary Figure 2 for the tumor volume bar plot. **(i)** Nerve injury (axotomy) experiment design; biweekly anti-mouse PD-1 treatment was administered 7 days after tumor implantation. **(j)** Tumor growth plot (day 39) of the axonotomy mouse experiment; Please refer to Supplementary Figure 2 for the tumor volume bar plot.

To evaluate the potential role of TANs in clinical response to anti-PD-1 therapy we used tumor samples from 55 patients with stage II-IVA cSCC who were enrolled in two clinical trials (NCT03565783 and NCT04154943). All patients underwent neoadjuvant anti-PD-1 therapy with Cemiplimab (Regeneron Pharmaceuticals) followed by surgery (Figure 1a, see baseline and neoadjuvant-treated sample distribution in Supplementary Figure 1). None of the patients underwent radiation treatments prior to the anti-PD-1 therapy. All patients received at least two cycles of Cemiplimab. In one trial (NCT04154943), the patients were allowed to receive up to 4 cycles of neoadjuvant Cemiplimab if they did not progress radiologically or clinically and tolerated the treatment^31^ (Figure 1b). Responders (n=31) were defined as patients with less than 10% viable tumor cells at surgery; non-responders (n=16) were defined as patients with more than 50% viable tumor cell in the neoadjuvant-treated surgical specimens, as previously described^31,32^. Patients who had 10%–50% viable tumor cells in the surgical specimens (n=8) were excluded from our cohort a-priori^31,32^ since this patient population has been inconsistently assigned to both the responders and non-responders groups in previous neoadjuvant clinical trials^33–36^. Some patients received adjuvant standard of care treatments after surgery, based on the judgement of the treating physician^31^.

Our first step in examining the potential role of TANs in clinical response to anti-PD-1 therapy was to assess tumors for the presence of PNI, as PNI is the most established and clinically relevant form of cancer-nerve interaction^37^. At baseline, non-responders had a significantly higher incidence of PNI compared to responders (71% versus 20%, respectively, p=0.041, Figure 1c). The definition of PNI is not based on functional evidence of nerve damage – PNI is a histo-morphological phenomenon, defined as the presence of tumor cells abutting or in close proximity to a nerve with encirclement of at least a third of the nerve circumference by tumor; or the presence of cancer cells within the epineurial, perineurial, and/or endoneurial compartments of a nerve^18^. To test whether cancer cells can damage the invaded nerves, we quantified the expression of canonical nerve response to injury markers ATF3 and c-Jun^38,39^ in the neural niches (Figure 1d). Analysis of baseline tumor samples (responders n=7, non-responders n=6, Figure 1e) revealed higher expression levels of both Schwan (i.e., GFAP^+^) c-Jun and neuronal (i.e., GFAP^−^) ATF3 (p=0.041 and p=0.005, respectively) in non-responders compared to responders. Others have demonstrated that TANs in close proximity to cancer cells were characterized by transcriptional alterations associated with nerve damage^40^ (Supplementary Table 1). These transcriptional alterations were over-expressed in neoadjuvant treated tumors of non-responders compared to responders (FDR 0.014, Figure 1f).

To test whether TAN damage may promote resistance to anti-PD-1 therapy, we used two neuromodulated cSCC mouse models^41^. First, we eliminated nerves from the TME by excising and plucking the nerves innervating the skin of immunocompetent SKH1-Elite (SKH1*-Hr*^*hr*^, Charles River^41^) mice. This procedure, called denervation, was done while preserving skin vasculature, and absence of nerves from the skin was confirmed by histology. Sham surgery was performed in the control group (Figure 1g). Skin denervation was confirmed one week post-denervation using behavioral testing^41^. SCC cells (B6, ultraviolet induced, SKH1*-Hr*^*hr*^ derived^42^) were orthotopically injected to the denervated skin. Seven days after cancer inoculation, mice were treated with either anti-PD-1 or IgG2 control. Denervated mice demonstrated improved tumor response to anti-PD-1 therapy with significantly lower tumor volumes compared to the control groups (*P* = 0.03, Figure 1h, Supplementary figure 2a). Next, we sought to validate the potential impact of TAN damage on response to anti-PD-1. Nerve damage was induced using surgical axotomy (Figure 1i). In this mouse model, severed nerves are left in place^41^, resulting in Wallerian degeneration^43,44^ (anterograde disintegration of axons and their transected myelin sheaths). One-week post-axotomy, cutaneous B6 SCC cells were orthotopically injected into the numb dermatome, followed by treatment with anti-PD-1. Axotomized mice had a significantly worse tumor control compared to sham control mice (*P* = 0.036, Figure 1j, Supplementary figure 2b). To confirm our findings and assess potential drug-specific effects of Cemiplimab on human immune cells, we repeated this experiment with human leukocyte antigen-matched human cutaneous SCC cells IC8^45^ that were injected into axotomized or sham operated skin of humanized CD34^+^ NOD-*scid* gamma (huCD34-NSG) mice. Initially both groups demonstrated response to Cemiplimab, however, only sham operated mice had durable response (417.7mm^3^ versus 105.4mm^3^ in axotomized and sham operated mice, respectively, *P* = 0.057 on day 40, Supplementary figure 2c-e).

### Cancer cells damage nerves by inducing nerve demyelination and degeneration

**Figure 2.**
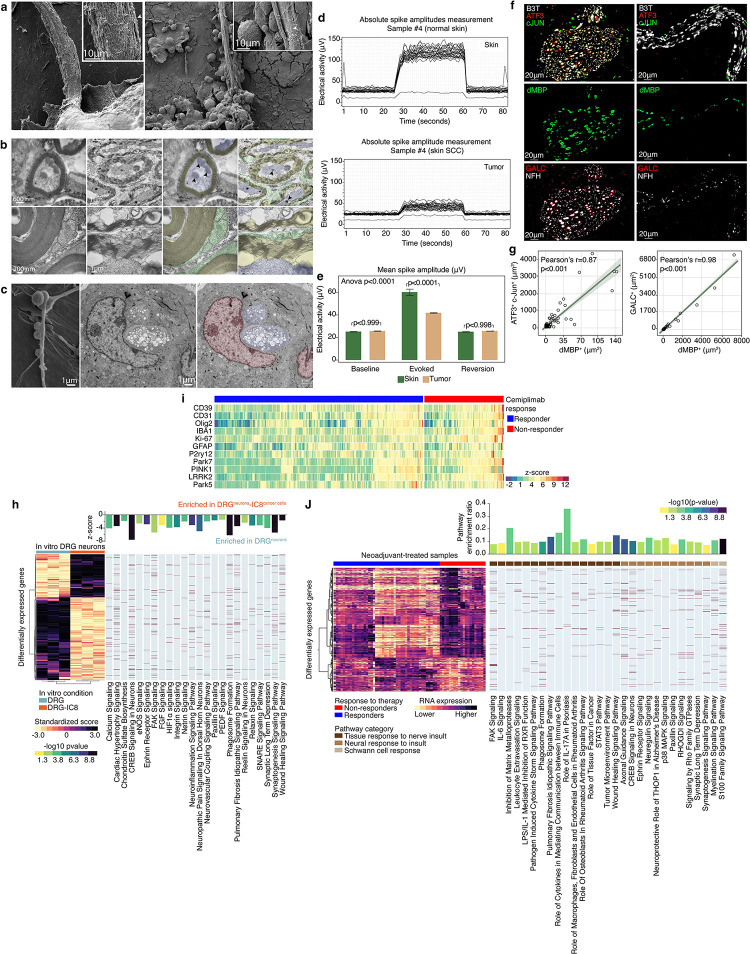
Cancer cells induce nerve demyelination and degeneration **(a)** Scanning electron microscopy (EM) images showing a naïve dorsal root ganglia (DRG) neuron (left, inset, x50,000) with normal myelin sheath, compared to a DRG neuron that was co-culture with squamous cell carcinoma cells (SCC, round cells, right image) for 5 days; The co-cultured neuron is invaded by the SCC cells and the myelin sheath has been degraded to circular and fibrillar fragments (right, inset, x50,000). **(b)** Transmission EM images – low-power field images are shown in the top row and high-power field images in the bottom row. In the first column, naïve neurons demonstrate compact myelin lamella; in the second column, DRG neurons which were co-cultures with SCC cells for 5 days demonstrated disintegration of the myelin sheath; The third and fourth columns show the same pseudocolored images with the myelin and Schwann cells in yellow and green, respectively. Arrowheads label mitochondria; note the dense abnormal mitochondria on the right, which together with dmyelination indicate axonal degeneration. **(c)** EM images demonstrating invasion of cancer cells (red) to the nerve inner layers (nerve filaments - purpule; first column – scanning EM, second and third columns – transmission EM). **(d-e)** Multielectrode array recordings of normal skin (control) and cutaneous SCC showing similar baseline and reversion electrical activity with blunted evoked response in tumor specimens – electrical conduction plot in **(d)** showed a single cancer-normal skin match, while the bar plots at **(e)** showed the mean ± SEM values of the entire group (n = 5–7 per group). **(f)** immunofluorescence (IF) stains of tumor samples from an indepdented cutaneous SCC patient cohort (n = 86; see main text for further details). B3T, beta-3-tubulin, a general nerve marker; cJUN and ATF3, markers of nerve damage; dMBP, degraded myelin base protein, and GALC, galactosylceramidase, are both markers of demyelination. **(g)** Pearson’s correlation plot between markers of nerve damage and de-myelination, based on the IF stains described above. **(h)** Transcriptional differences in DRG neuron that were co-cultured with IC8 SCC cells (DRG-IC8) compared to DRG neurons alone; data presented in a heatmap structured by unsupervised hierarchical clustering analysis (HCA); the enriched pathways are based on Ingenuity Pathway Analysis (IPA) and their respective z-scores and p-values (Fisher’s Exact test) are shown in the adjacent chart, together with the differentiatly expressed genes (DEG) of the depicted pathways (highlighted in brown). All experiments were done in biological triplicates. **(i)** The heatmap shows the protein expression of neurodegeneration-associated markers enriched within intra-tumoral neural niches of non-responders patients compared with responders. Protein expression was measured by digital spatial profiling (DSP) and transformed into z-scores for representation. **(j)** Heatmap showing transcriptional differences between responders (R) and non-responders (NR) in neoadjuvant-treated tumor samples and the corresponding enriched pathways. See Extended figure 1 for number of samples in each analysis.

To decipher the mechanism of TAN damage, we examined the interaction between SCC cells and neurons in vitro. Freshly harvested murine dorsal root ganglia (DRG) neurons were kept intact to maintain the integrity of the explant and prevent compromise of the cell-cell contact between neurons, Schwan cells, and endoneurial macrophages^46,47^. DRG neurons were co-cultured with murine SCC cells (Moc1 and B6). As seen in Video 1, the SCC cells were neurotropic, and within 72 hours made direct contact with the axon. The ultra-structural changes associated with the direct cancer-neuron contact were assessed using electron microscopy (EM). Scanning EM images were obtained on day 5 of the co-culture and confirmed the cancer cell attachment and invasion to the epineurium (Figure 2a). Compared to naïve neurons, co-cultured neurons demonstrated impaired myelin integrity. The myelin debris presented as large circular aggregates, which were strikingly distinct from the linear appearance of normal compact myelin (Figure 2a). On transmission EM, disintegration was evident at the endoneurial and axonal level, with disentanglement of the compact myelin lamellae (Figure 2b). After seven days of co-culture, a complete loss of the myelin sheath was found at the point of contact with cancer cells, as well as proximal loss of compact myelin integrity and axonal mitochondria – implying the presence of retrograde Wallerian degeneration (Figure 2c).

Myelin degradation is associated with decreased nerve conduction velocity and propagation of evoked electrical activity^48^. To provide in vivo functional evidence of cancer induced myelin degradation, we performed high-throughput electrical conduction studies using murine cSCC model. Using multi-microelectrode array (MEA), electrophysiological recordings were obtained from 3–5 mm orthotopic cSCC (intradermally injected B6 cells) and from non-tumor bearing healthy control skin (Figure 2d). Simultaneous spatial and temporal continuous recording of the extracellular field potential (FP) revealed that at baseline, spontaneous potentials of tumor bearing skin was comparable with normal skin. However, upon stimulation, the evoked electrical activity in tumor bearing skin was significantly lower than the activity in normal skin (41.8μV and 60.2μV,respectively, p<0.0001, Figure 2d) with significantly increased thresholds. Reversion (“back to baseline”) potentials were comparable (Figure 2e), but tumor bearing skin demonstrated compound potentials, suggestive of demyelination^49^.

To confirm that the mechanism driving cancer induce nerve damage is de-myelination, multiplex immunofluorescence stains were conducted on tumor samples from an independent validation cohort of 86 treatment naïve cSCC patients. This cohort included patients with localized (T1-3^50^) disease who underwent Mohs surgery at the University of Texas MD Anderson Cancer Center. This external cohort was used to test our hypothesis that cancer induced nerve damage occurs early in the disease course and represent an intrinsic cancer cell trait, rather than a marker of advanced disease. Tissue sections were stained for general nerve markers (beta-3-tubulin, B3T), markers of nerve damage (cJUN and ATF3), and markers of de-myelination (degraded myelin base protein, dMBP, and galactosylceramidase, GALC, Figure 2f). We found a significant correlation between nerve insult (ATF3^+^cJUN^+^) and de-myelination (dMBP+, pearson’s correlation co-efficient=0.87 p<0.0001, Figure 2g). Due to the proximity of blood vessels to nerve in the TME (neurovascular bundles), we sought to rule out a vascular injury that might contribute to the nerve damage. Immunohistochemistry stain against ERG, a marker for endothelial cells, revealed that nerve damage was not associated with a vascular injury (Supplementary Figure 3). These findings further confirmed that TAN damage is associated with peripheral demyelination.

Demyelination is a hallmark of central neurodegenerative diseases, such as Parkinson’s disease, Alzheimer’s disease, and amyotrophic lateral sclerosis^51–53^. Hence, we sought whether transcriptomic pathways associated with these central neurodegenerative diseases might be present in peripheral nerves exposed to cancer. Freshly harvested human DRG neurons were co-cultured with human cSCC cells (IC8^45^) for 5 days. Cells were sorted, and NeuO^+^ cells (live neurons) underwent RNA sequencing. Compared to a neuron-only controls, neurons that were co-cultured with cancer cells significantly downregulated genes involved in homeostasis, neuronal repair, and neuronal survival pathways, including the CREB pathway, FAK signaling, synaptogenesis, phagosome formation, calcium signaling, and SNARE complex, FDR < 0.01, Figure 2h). To assess for potential direct effect of the anti-PD-1, human DRG neurons were co-cultured with cSCC cells with and without anti-PD-1 antibodies (Cemiplimab, Regeneron). DRG neurons co-cultured with cancer and anti-PD-1 had similar transcriptional profile compared to DRG neurons co-cultured with cancer without anti-PD1 (58 differentially expressed genes out of 25,688 overall identified genes, 0.2%, Supplementary Figure 4a, Supplementary Table 2). Since tumors in the head and neck region are innervated by the trigeminal ganglia (TG), this experiment was repeated using murine TG neurons co-cultured with murine cSCC B6 cells. The results for the murine TG model were similar to those of the human DRG model – downregulation of multiple canonical nerve homeostatic pathways, including neuronal repair, synaptogenesis and neuroinflammatory pathways in TG neurons co-cultured with cancer cells compared to TG alone (Supplementary Data Figre 4b-d). Overall, these findings suggested that direct interaction between cancer cells and neurons result in cancer associated peripheral nerve degeneration (CAPND).

Next, we assessed for evidence of CAPND in our human cSCC clinical trials cohort. The degeneration-regeneration homeostatic status of TANs was assessed via NanoString GeoMx Digital Spatial Profiler (DSP). The protein neuron profiling panels included markers of neural degeneration (e.g., α-synuclein, LRRK2 and Park5/7) and neuro-inflammation (e.g., IBA1 and TMEM119). Neural niches were identified via immune labeling of the nerve morphology markers neurofilament heavy chain (NFH) and B3T. A total of 553 neural niches (region of interests, ROI, *n* = 84 for baseline samples, *n* = 469 for neoadjuvant-treated) were identified and analyzed using the nCounter protein expression analysis system (Supplementary Figure 5a-d). The expression of neurodegenerative proteins in neoadjuvant-treated samples of non-responders (ROI n=109) was compared to those of responders (ROI n=360). Tumors from non-responding patients exhibited increased protein expression of PARK7 (FDR < 0.0001), PARK5 (FDR = 0.01), PINK1 (FDR = 0.006), and LRRK2 (FDR < 0.0001), as well as activation and proliferation markers of Schwann cells such as P2RY12 (FDR = 0.001), OLIG2 (FDR = 0.01), and GFAP (FDR = 0.009 Figure 2i) ^54^. Analysis of the baseline tumor samples showed that the neuro-protective protein APOA-I (FDR = 0.01) and the neuronal repair microglia marker TMEM119 (FDR = 0.01) were over-expressed among responders versus non-responders (Supplementary Figure 5e). The DSP protein data was validated by bulk tumor RNA sequencing analysis. Ingenuity Pathway Analysis (IPA) of neoadjuvant-treated samples showed that non-responders significantly up-regulated pathways associated with neural response to injury, such as CREB signaling in neurons, FAK signaling, synaptic excitability, phagosome formation, and myelination neuroprotective role of THOP1 in Alzheimer’s disease (FDR <0.01, Figure 2j). Analysis of baseline samples showed that compared to responders, non-responders significantly downregulated LXR/RXR, RHOGDI signaling, and antioxidant action of ascorbic acid – all needed for proper nerve recovery and conclusion of the repair processes after nerve damage ^43,55–57^ (all FDR <0.01, Supplementary Figure 5f). Overall, these findings suggested that non-responders to anti-PD-1 therapy had a higher degree of CAPND in both their neoadjuvant-treated and baseline samples.

### Cancer associated peripheral nerve degeneration (CAPND) correlates with an inflammatory and tumor promoting TME

**Figure 3.**
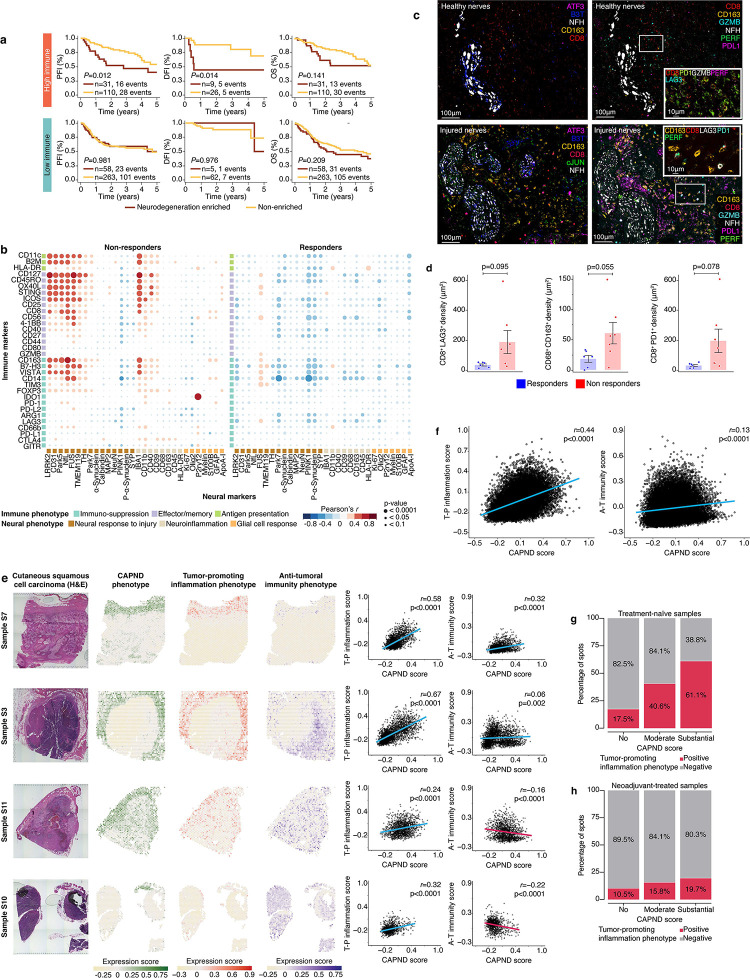
Cancer associated peripheral nerve degeneration (CAPND) induces tumor promoting inflammation **(a)** Kaplan-Meier analyses showing progression-free interval (PFI), disease-free interval (DFI), and overall survival (OS) plots for a head and neck mucosal squamous cell carcinoma (SCC) patient cohort from the TCGA database. Analysis was stratified according to the intra-tumoral immune activity score^58^. The patients were further stratified according to their CAPND enrichment score (see Supplementary Figure 6) – CAPND high (red) and CAPND low (yellow) patients. Log-rank test, see text for adjusted p values. **(b)** Bubble heatmap based on digital spatial profiling (DSP) protein matrix. The heatmap shows Pearson’s correlation coefficients between immune and neural proteins expressed specifically in the peri-neural niche of of neoadjuvant-treated tumor samples of the clinical trial cohort. **(c)** Multiplex-immunofluorescence (IF) stains of healthy (NFH^+^B3T^+^ATF3^−^, top panels) and damages (ATF3^+^cJUN^+^, bottom panels) tumor-associated nerves (TANs). The area around the nerves (i.e., peri-neural niche) was stained for CD163 (tumor associated macropahges), CD8 (effector T-cells), LAG3 and PD1 (immune checkpoints), and Gramzyme B and perforin (cytotoxic proteins). **(d)** Bar plots representing IF-based cell density according to clinical response to anti-PD-1 therapy. **(e-g)** Spatial transcriptomic analysis of tumor samples fron an independent treatment naïve cutaneous SCC patient cohort (see main text for full details). This analysis assessed the co-localization of three phenotypes: CAPND, tumor-promoting inflammation, and anti-tumoral immunity (55μm resolution per spot). The signature scores (normalized and zero-centered) obtained for each spot were overlaid on their respective tissue region, showing the proportional content and spatial distribution and overlap of the different phenotypes. The correlation between CAPND, the tumor-promoting(T-P) inflammation, and the anti-tumoral (A-T) immunity phenotypes per sample is shown on the right **(f)** Similar correlation plots to those of (e), this time showing the overall correlation across the entire 11 patient cohort. **(g)** Stacked bar plots representing sequenced tissue spots grouped according to their CAPND phenotype score – Substantial (defined as fold change >2 with FDR < 0.01), Moderate (defined as fold change 0–2, FDR <0.01), and no (fold change <0). The red bars represent the ratio of spots with positive tumor promoting inflammation phenotype scores (defined as fold change >2 with FDR < 0.01) out of all of the assessed spots, for each CAPND group. This bar plot is demonstrating a higher ratio of CAPND and inflammation co-localizion among areas of high substantial CAPND scores. **(h)** Similar stacked bar plot and spatial analysis conducted on netoadjuvant-treated samples from our clinical trial cohort (n = 16).

To validate the correlation between CAPND and poor clinical outcome, we assessed the expression of neurodegeneration-related gene pathways in SCC samples from The Cancer Genome Atlas (TCGA). Since a cSCC patient cohort was not available at the TCGA database, we used a cohort of mucosal head and neck SCC (HNSCC). HNSCC is a different cancer type than cSCC, hence this TCGA analysis served as an external validation. The TCGA analysis focused on HNSCC patients with stage II-IVa (*n* = 462), as these were the stages of our clinical trial cSCC patient cohorts. A CAPND signature was created based on neurodegeneration pathways that were enriched in our cSCC clinical trial cohorts (e.g., Alzheimer disease, Human Phenotype Ontology [HPO] M35868 axonal degeneration, HPO M38571; Supplementary Figure 6a). A CAPND enrichment score was calculated for each patient, indicating enrichment for neurodegenerative pathways. Of note, CAPND scores correlated with the presence of PNI (p = 0.0011, Supplementary Figure 6b) but not with disease site, stage and HPV status. Among the TCGA HNSCC patients, 90 had a high CAPND score, while 373 patients had a low CAPND score. Compared with low CAPND score patienths, high score patients had a shorter disease-free interval (*P* = 0.016) and a trend toward worse overall survival (P=0.09, Supplementary Figure 6c). Next, we stratified the TCGA HNSCC cohort into two groups based on an anti-tumoral immune signature which was previously associated with clinical response to anti-PD-1 therapy in HNSCC patients^58^ (Supplementary Figure 6d). Among patients with high anti-tumoral immunity score (Figure 3a), a high CAPND score was associated with worse disease-free survival (P = 0.014) and progression free survival (P = 0.012), but not with overall survival. The correlation of CAPND with adverse disease free and progression free survival remained statistically significant after adjusments for clinical variables such as age, HPV status, site, and staging (results of both univariant and multivariant analyses were presented in Supplementary table 3). CAPND score did not correlate with survival in patients with low anti-tumoral immunity. These TCGA findings suggested that CAPND can impair the activity of an existing intra-tumoral immune infiltration.

Following a peripheral nerve injury, neurons and Schwan cells attract immune cells to the peri-neural niche to initiate an inflammatory response aimed at nerve healing and regeneration^44^. Hence, we hypothesized that CAPND was associated with the presence of pro-inflammatory, tumor promoting immune activity. To test this hypothesis, we assessed potential differences in the peri-neural niche immune activity between responders and non-responders. This architectural analysis was done using the DSP protein expression data. Peri-neural niches of neoadjuvant-treated non-responders showed correlation between markers of neuronal response to injury and various immune markers, including immune makers associated with tumor progression such as CD163 (tumor associated macrophages), FOXP3 (T regulatory cells, Tregs), and the immune checkpoints VISTA and IDO-1 (Figure 3b). In contrast, peri-neural niches of responders showed mainly an inverse correlation between markers of neuronal response to injury and immune markers. These findings were validated using multiplex immunofluorescence stains of the peri-neural niches (Figure 3c). Analysis of the peri-neural niches (defined as an area within 150 μm from the epicenter of TANs^59^) in neoadjuvant-treated samples showed that CD68^+^CD163^+^ cells, as well as CD8^+^PD1^+^ and CD8^+^LAG3^+^ cells (exhausted CD8^+^ T cells) were more abundant in non-responders compared to responders (p=0.055, p=0.078, and p=0.095, respectively, Figure 3d). Collectively, these findings suggested co-localization of a CAPND and an inflammatory, tumor promoting immune activity.

To assess the impact of CAPND on the global anti-tumoral immune activity, we performed spatial transcriptomic analysis on tumor samples from an independent, treatment-naïve cSCC patients who underwent surgery at The University of Texas MD Anderson (n = 11 patients; Supplementary Table 4). In this spatial analysis, we determined the co-localization status of three functional phenotypes: CAPND (expressing the nerve markers NEFL^+^NEFH^+^NEFM^+^NEUROD1^+^MRGPRD^+^TAC1^+^SSTR2^+^HAPLN4^+^SST^+^; and positive for nerve damage markers ATF3, JUN, SOX1, SMAD1, BHLHE41, KLF7, or KLF6 ^43^), anti-tumoral immunity (CD8A^+^GZMB^+^PRF1^+^ and CD4^+^IL2^+^ T cells; and CD86^+^IRF8^+^TNF^+^ and CD68^+^PSMB10^+^HLADQA1^+^HLADRA^+^HLADRB1^+^ antigen presenting cells), and tumor-promoting infalmmation (CD204^+^CD206^+^CD163^+^ and CD68^+^IL-10^+^ tumor-associated macrophages; and CD4^+^FOXP3^+^ T regulatory cells). Figure 3e showed the spatial distribution of the CAPND, tumor-promoting infalmmation, and anti-tumoral immunity phenotypes in individual tissue sections and the respective correlation between the CAPND and immunity scores. In this treatment-naïve cohort, the CAPND phenotype correlated with the tumor-promoting infalmmation phenotype (R=0.44) but not with the anti-tumoral immunity (R=0.13, Figure 3f). Analysis of 27,420 tumor regions revealed that 61.1% of the regions with substantial presence of the CAPND phenotype (defined as fold change >2 with FDR < 0.01) co-localized with the tumor-promoting infalmmation phenotype (n = 1544 of 8812 and 4780 of 7817, p < 0.001 Figure 3g).

Next, we validated these spatial findings in the cSCC clinical trial cohort. Among the neoadjuvant-treated tumors, region with CAPND phenotype co-localized the tumor-promoting inflammation phenotype higher compared to regions without CAPND (n = 688 of 6571 and 596 of 3019, p < 0.001 Fig. 3h). To further validate these finding, a similar spatial transcriptomic analysis was conducted on tumors derived from our nerve injury mouse hSCC model (see above) treated with the Cemiplimab. The CAPND phenotype was enriched among axotomized mice compared with sham operated mice (Supplementary Figure 7a). These enriched regions were spatially associated with increased tumor-promoting inflamatorry activity in axotomized mice compared to sham operated mice, but not with the anti-tumoral immunity phenotype (Supplementary Figure 7b). Taken together, these results suggested a functional role for CAPND in facilitating an inflammatory, tumor-promoting immune activity that affect the general TME immune tone and hence dampen the clinical efficacy of anti-PD-1 therapy.

### Blockade of TAN-induced inflammatory signals enhanced anti-PD-1 efficacy

**Figure 4.**
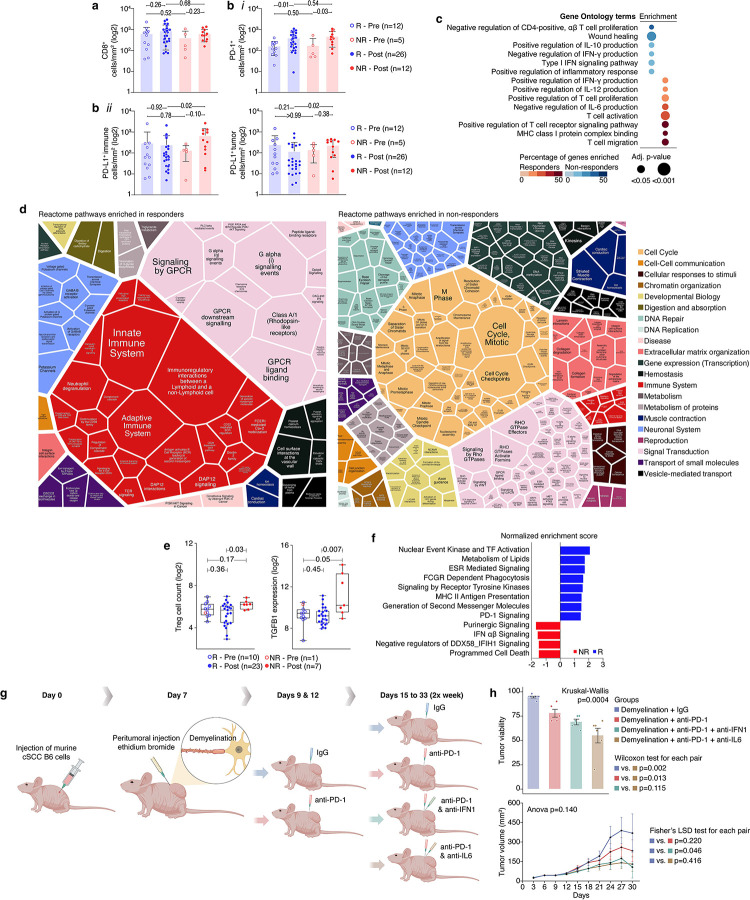
Cancer associated peripheral nerve degeneration (CAPND) induces sustained pro-inflammatory signaling which functionally impairs anti-tumoral immune activity **(a)** Immunohistochemically (IHC) based cell count of CD8+ cells in the clinical trial cohort, according to the clinical response status to anti-PD-1 therapy; R, responders; NR, non-responders; Pre – baseline tumor samples; Post, neoadjuvant-treated samples. **(b)** Similar IHC-based cell count of (i) PD-1^+^, (ii) PD-L1^+^ immune cells, and PD-L1^+^ tumor cells. **(c)** Gene Ontology (GO) enrichment analyses of bulk tumor RNA sequencing (clinical trial cohort) demonstrating the different immune landscape in neoadjuvant-treated tumor sample of responders (red) and non-responders (blue) **(d)** Voronoi treemaps based on Reactome gene pathway analysis of bulk tumor RNA sequencing (clinical trial cohort), providing an overview of the pathways that were enriched among responders and non-responders; The colors represent the parent pathways (legend) associated with each enriched term. **(e)** Nanostring nCounter PanCancer analysis of the clinical trial tumor samples showing T regulatory cells (Tregs) and Tumor Growth Factor (TGF)-ß1 expression based on response at base line and on treatment. **(f)** Nanostring nCounter PanCancer pathway enrichment analysis of neoadjuvant-treated samples according to response status. The presented pathways were considered significantly enriched at FDR < 0.2. NES, Normalized Enrichment Scores. Baseline sample analysis was not done due to a small avaialbe samples size (baseline non-responder n=1). **(g)** De-myelination and inflmmatory signal blockade mouse experiment design; a de-myelinating agent (ethidium bromide; EtBr has no short term mutagenic effects) was injected to the periphery of cutaneous squamous cell carcinoma (SCC) tumors. The mice were then treated with IgG control, anti-PD-1 monotherapy, or combination of anti-PD-1 with either IFN-I receptor or interleukin(IL)-6 blockade. IFN-I and IL-6 were selected as targets based on the described above gene pathway analysis. **(h)** Tumor growth and tumor viability plots of the different groups in the in demyelinated mouse experiment (p values represent ANOVA, and post hoc Fishers Least Significant Difference tests).

To further validate the expansion of pro-nerve healing, tumor-promoting inflammation from the per-neural niche to the rest of the TME, we profiled intra-tumoral immune difference between responders and non-responders from our clinical trials cohort. Immunohistochemical staining of tumor samples demonstrated no differences in CD8^+^ T-cell abundance between responders and non-responders either before or after treatment (Figure 4a). Since CD8^+^ T-cells could properly infiltrate tumors of non-responding patients, we hypothesized that these T-Cells encountered a hostile TME, leading to their functional impairment. To test this hypothesis, we first stained for PDL-1, since PDL-1 acts as a negative feedback loop suppressing CD8+ T-cell activation^60,61^. Neoadjuvant-treated tumors of non-responders had significantly higher expression of PD-L1 on both tumor cells and immune cells compared to responders (p=0.02 and p=0.02, respectively, Figure 4b). Next, gene pathway analysis was performed on bulk tumor RNA to assess for functional immune differences. GO pathway analysis of baseline tumor samples did not identify any immune-related pathways that significantly differed according to response status. GO pathway analysis of neoadjuvant-treated tumor of responders demonstrated upregulation of antitumoral immune-related pathways (Figure 4c), such as upregulation of IL-12 production (GO:0032735; FDR < 0.001), T-cell activation (GO:0042110; FDR = 0.001), and positive regulation of IFN-γ production (GO:0032729; FDR = 0.03). In contrast, non-responders upregulated tumor-promoting pathways, such as negative regulation of IFN-γ production (GO:0032689; FDR < 0.01), positive regulation of IL-10 production (GO:0032733; FDR = 0.03), and wound healing (GO:0042060; FDR < 0.01). A Voronoi heatmap based on Reactome RNA pathway analysis emphasized the major role of immune-related pathways among responding tumors (Figure 4d and Supplementary Figure 8). Interestingly, type I interferon signaling pathway (GO: 0060337) was up-regulated among neoadjuvant treated non-responders (Figure 4c). This observation was confirmed via NanoString nCounter PanCancer Immune Profiling Panel. The Nanostring analysis demonstrated a higher Treg infiltration and a higher tumor growth factor (TGF)-β−1 expression among neoadjuvant-treated non-responders (Figure 4e,). It also demonstrated upregulation of IFN-α and IFN-β (type I IFN, IFN-I) signaling in the tumors of neoadjuvant-treated non-responders (Figure 4f and Supplementary Figure 9). These findings initially seemed counterintuitive, as IFN-I signaling has been associated with a favorable response to immunotherapy^62–64^. However, while acute IFN-I signaling can promote antitumoral immunity, chronic IFN-I signaling has been shown to drive immunotherapy resistance^65,66^. To determine if CAPND can be the source of IFN-I signaling we returned to the in vitro model of DRG neurons cultured alone or with cSCC cells for 5 days (see above). RNA sequencing of the neurons from this model demonstrated that neurons co-culture with cancer cells up-regulated not only neurodegenerative pathways, but also IFN-I signaling pathways (Positive regulation of type I interferon production, GO:0032481; FDR < 0.0001, and regulation of type I interferon production, GO:0032479; FDR=0.01). Moreover, cancer exposed neurons also up-regulated inflammatory pathways, such as positive regulation of IL-1β production (GO:0032731; FDR < 0.0001), and positive regulation of IL-6 production (GO:0032755; FDR < 0.0001, Supplementary Table 5).

To confirm that CAPND is indeed the source of chronic IFN-I activity among non-responders, we sought other, non-CAPND related, potential sources for activation of IFN-I signaling. As the microbiome has been previously associated with IFN-I signaling^67^, we analyzed the tumor-associated microbiome (including analysis of the bacteriome, virome, and mycobiome [fungi]) in our cSCC clinical trial samples. We observed no differences in the intra-tumoral microbiome composition between responders and non-responders (Supplementary Figure 10 and Supplementary Table 6). Another potential driver of the differential response to anti-PD-1 therapy is tumor mutational burden^68^. However, a previous report failed to show an association between tumor mutational burden and clinical response among cSCC patients undergoing neoadjuvant anti-PD-1 treatment^31^.

Finally, we sough to reverse the CAPND-derived resistance to anti-PD-1 therapy via blockade of inflammatory signals in vivo (Figure 4g). One week after injection of B6 cSCC cells to 36 mice, a demyelinating agent (ethidium-bromide) was injected to the tumor periphery at the tumor-normal skin interface,as previously described^69–71^. Anti-PD-1 treatment was initiated two days after the demyelination. To allow priming of immune cells against the tumor^72^, the first two anti-PD-1 doses were administered alone. Subsequent anti-PD-1 doses were given alone, or in combination with antibodies blocking either interferon-α-receptor-1 or IL-6. The addition of anti-inflammatory antibodies did not reduce the tumor size in a statistically significant manner (Figure 4h). However, in both of the neoadjuvant Cemiplimab clinical trials, there was no correlation between the tumor size per imaging to pathological response to treatment^31,32^, with 51% pathological CR rate versus only 6% of radiological CR^31^. Hence, we assessed the pathological response to treatment in our mice model. The addition of anti-IL-6 to anti-PD-1 in the setting of TANs demyelination had significantly improved treatment efficacy, with an average of only 55% viable tumor cells versus 78% viable tumor cells in the anti-PD-1 monotherapy arm (Figure 4h).

## Discussion

Although the role of the nervous system in immune regulation has gained recognition in recent years^16,73^, little is known about the ability of the PNS to modulate anti-tumoral immune activity. Our multi-omics data from tumors of patients who underwent neoadjuvant anti-PD-1 therapy suggested that TANs can promote clinical resistance to anti-PD-1 therapy. We proposed a mechanism driving this clinical effect. Cancer cells cause demyelination of nerves, as evident by structural changes of myelin and abnormal nerve electrical conduction studies (Figure 2). This demyelination results in nerve damage and degeneration. Transcriptomic and protein profiling showed that the neurons and their Schwan cells attempted to regenerate and heal the nerves. The nerve healing process requires recruitment of immune cells to the peri-neural niche^44^, leading to peri-neural inflammation. This inflammatory response is characterized by secretion of IL-10 and TGF-β, polarization of macrophages to an M2 subtype, and infiltration of Tregs^44,46,74–77^ – all are known suppressors of anti-tumoral immunity. The degraded myelin fragments are known damage-associated molecular patterns (DAMPs) molecules, capable of stimulating an IFN-I response by cells in the peri-neural niche^78^. In parallel to the continuous damage inflicted by cancer cells, the IFN-I signaling by itself can propagate the nerve degeneration process^79–81^ and promote the cycle. While acute IFN-I signaling has a potent anti-tumoral effect, a continuous IFN-I signaling can suppress anti-tumoral activity^82^. Continuous IFN-I signaling attracts Tregs to the TME, increases PDL-1 expression on dendritic cells, and exhausts effector T-Cells^83,84^. These inflammatory changes, the expansion of inflammation from the peri-neural niche to the rest of the TME, and the consequential functional impairment of intra-tumoral CD8^+^ T-Cells were found in tumors of anti-PD-1 non-responders from our clinical trials cohort. Hence, our findings suggest a new anti-PD-1 resistance mechanism which is mediated by damaged TAN and their associated inflammation. This resistance mechanism may be relavent to other, non-cSCC neurotropic cancer with an overall poor response to anti-PD-1 therapy such as pancreatic^85^, prostate^86^, and breast^87^ cancers.

A key finding of our study is that the TAN-derived anti-PD-1 resistance may be clinically targetable and reversible. Our murine model results demonstrated that combined blockade of PD-1 and the pro-inflammatory cytokine IL-6 improved anti-PD-1 efficacy (Figure 4). While our results are preliminary, blocking inflammatory signaling to enhance anti-PD-1 clinical efficacy is an exciting and rapidly evolving field, which is already being tested in metastatic melanoma and non-small cell lung cancer patients (NCT04940299, NCT03999749). As another potential therapeutic approach, the inflammatory signaling might be blocked by addressing its root cause – nerve degeneration. Neuroprotective agents may, theoretically, dampen CAPND. Moreover, markers of nerve degeneration may serve as future bio-markers to identify patients with lower chances of responding to anti-PD-1. While the current study did not provide evidence in humans for the efficacy of such treatments or biomarkers, it is among the first to introduce the concept of TAN-derived modulation of anti-tumoral immunity, hence supporting future research in this field.

A major limitation of this study is the fact that different patient-based analyses had different sample sizes (Supplementary Figure 1). The number of samples from our cSCC clinical trial was limited. Hence, some analyses had fewer available tissues, which could have impaired their statistical power. Another limitation is a higher number of neoadjuvant-treated samples compared to the number of baseline samples. Since this was a neoadjuvant trial, all patients underwent surgery after the neoadjuvant treatment and had available tumor tissue for analysis. Moreover, since the entire tumor was removed during surgery, some patients had several neoadjuvant-treated tumor samples collected in order to capture the heterogenicity of the tumor. In contrast, not all patients underwent a baseline tumor biopsy^31^. While the number of clinical samples was limited, our cohort represents patients with clearly defined disease, treatment, and sample collection time points – enabling us to relate molecular changes to clinical responses. Moreover, the extensive in vitro and in vivo analyses, based on multiple different methodologies, validated the clinical samples based observations.

In conclusion, our findings revealed a potentially new resistance mechanism to anti-PD-1 therapy derived by TANs damage and degeneration. These insights might aid in identifying novel biomarkers and developing novel therapeutic agents targeting specific features of nerve degeneration or its associated tumor-promoting inflammation. Combined with immunotherapy, such agents may improve patients care across different tumor types.
